# A comparison of emotion-focused therapy and cognitive-behavioural therapy in the treatment of generalised anxiety disorder: study protocol for a randomised controlled trial

**DOI:** 10.1186/s13063-018-2892-0

**Published:** 2018-09-19

**Authors:** Ladislav Timulak, Daragh Keogh, Craig Chigwedere, Charlotte Wilson, Fiona Ward, David Hevey, Patrick Griffin, Louise Jacobs, Brenda Irwin

**Affiliations:** 1 Trinity College Dublin, Dublin, Ireland; 2 HSE National Counselling Service, Naas, Ireland

**Keywords:** Generalised anxiety disorder, Emotion-focused therapy, Cognitive behavioural therapy, Primary care

## Abstract

**Background:**

Generalised anxiety disorder (GAD) is a chronic and debilitating condition characterised by high co-morbidity. Alongside pharmacological treatment, cognitive behavioural therapy (CBT) is an established psychological therapy for GAD. Its effectiveness is limited, however, with only an estimated 50% of clients presenting in the non-clinical range after a course of treatment. Furthermore, not all clients prefer CBT as a psychological therapy. Recently, emotion-focused therapy (EFT) was developed for GAD and was tested in an open trial with promising results.

**Methods/design:**

The present research project is a feasibility testing randomised controlled trial (RCT) that compares the efficacy of EFT with an established treatment for GAD, CBT. Sixty clients presenting in a primary care psychology/counselling service will be randomly assigned to one of two conditions: EFT or CBT. Outcomes will be assessed using several measures (Generalised Anxiety Disorder-7, Generalised Anxiety Disorder Severity Scale, Patient Health Questionnaire-9, and Clinical Outcome in Routine Evaluation – Outcome Measure). Clients will be assessed prior to and at the end of therapy, as well as at 6-month follow-up. On the basis of findings from the initial open EFT trial with regard to the optimal length of therapy, it is proposed that therapy last between 16 and 20 sessions.

**Discussion:**

This study aims to test the feasibility of a full comparison RCT. It will test subject recruitment, therapist adherence to manualised treatment, and client retention rates. It will also provide estimates of comparative outcomes that can inform power calculations for a definitive trial.

**Trial registration:**

ISRCTN Registry, ISRCTN52689081. Registered on 24 October 2017.

**Electronic supplementary material:**

The online version of this article (10.1186/s13063-018-2892-0) contains supplementary material, which is available to authorized users.

## Background

Generalised anxiety disorder (GAD) is a common anxiety disorder [1]. Its 1-year prevalence is around 3%, whereas its lifetime prevalence is around 5% [2–5]. Furthermore, co-morbidity is high, with almost 60% of individuals diagnosed with GAD also meeting diagnostic criteria for depression and an additional anxiety disorder [6]. Co-morbidity with personality disorders is also very high, particularly avoidant and dependent personality disorders [7]. People with GAD experience significant impairment in quality of life comparable to that of people with depression [8, 9]. GAD represents a significant cost to society owing to disability, decreased work productivity and increased use of health care services [5].

Current guidelines recommend that GAD be treated by medication and/or a psychological therapy (cf. National Institute for Health and Care Excellence [NICE] [10]). Among psychological treatments for GAD, various forms of cognitive behavioural therapy (CBT) are the most recognised treatments [11]. CBTs for GAD have been shown to be more effective than wait-list comparisons [12–14]. CBT treatments for GAD include a number of common interventions; however, there is significant variation among them [11]. Broadly speaking, CBT models of treatment variously use a number of the following components/interventions: psychoeducation about worry, cognitive restructuring of beliefs relating to worry, problem solving, monitoring and shaping of the worry process, relaxation training, exposure, and behavioural experiments, and others [11].

Although the efficacy of CBTs is well studied, not all clients benefit from CBT. Less than 50% of clients recover by the end of the treatment, and long-term follow-up data is sparse [13, 15]. A Cochrane review of psychological therapies for GAD recommends that “further studies examining non-CBT models are required to inform health care policy on the most appropriate forms of psychological therapy in treating GAD” [14]. Furthermore, research on patient preferences for therapies for other disorders shows that some patients prefer other psychological therapies to CBT [16]. The development of new treatments for GAD may also show that the effectiveness of new treatments may differ when compared with CBT, as has been shown, for example, in the case of depression [17]. These arguments led a group of researchers to adapt emotion-focused therapy (EFT) for the treatment of GAD [18, 19].

EFT [20, 21] is a research-informed therapy that focuses on (1) increasing awareness of adaptive and maladaptive emotions, (2) enhancing emotion regulation, (3) transforming maladaptive emotions and (4) reflecting on adaptive emotions [22]. EFT is an empirically supported treatment for major depressive disorder [17, 23] and is also extensively studied for complex trauma [24]. In comparison with CBT for depression, EFT showed comparable results with a slight advantage for EFT in the domain of interpersonal functioning [17]. EFT is also currently being adapted for other mental health disorders, such as social anxiety, where the central problems lie in maladaptive emotional processing [25–28]. Because GAD is understood to be caused by difficulties in emotional processing (e.g., an incapacity to tolerate difficult emotions, combined with a tendency to avoid such difficult emotions) [11, 29], a therapy such as EFT that focuses on the processing and transformation of painful emotions is theoretically a good match for GAD difficulties [18]. Furthermore, EFT also focuses on the overcoming of emotional avoidance, a central feature of GAD.

A group of researchers, led by the lead author of the present article, developed EFT for GAD [18, 19] and tested it in an open trial with promising results [30]. The present project should further contribute to the exploration of EFT as a viable alternative to CBT in the treatment of GAD. The feasibility study should establish the relative efficacy of EFT in comparison to CBT as an established treatment.

### Objectives

The overall aim of the project is to contribute to the planning of a definitive non-inferiority trial that would establish the relative efficacy of EFT in comparison to CBT as an established treatment. The present project will provide first-comparison data that, if suggesting comparable or better outcomes, should help to plan a definitive non-inferiority trial. It will test recruitment, adherence, and retention rates, as well as provide estimates of comparative outcomes that can be used to inform power calculations for a definitive non-inferiority trial.

## Methods/design

### Trial design and setting

The design of the study is a randomised controlled trial (RCT) with two active parallel interventions (EFT and CBT). This reported version of the protocol conforms to the Standard Protocol Items: Recommendations for Interventional Trials (SPIRIT) guidelines. (For a copy of the SPIRIT checklist, *see* Additional file 1).

Participants will be recruited from a national public health primary care service in Ireland. The Health Service Executive (HSE) provides public health services across Ireland, and the HSE’s Counselling in Primary Care (CIPC) service provides counselling to clients who present to primary care with mental health difficulties such as anxiety and depression. CIPC was established by the HSE on a nationwide basis in 2013 following the commitment outlined in the Programme for Government strategy [31] to implement Vision for Change [32] and improve access to psychological therapy.

### Participants/clients

Participants (*n* = 60) will be adults (aged ≥ 18 years) who are medical card holders (i.e., entitled to free public health care) referred by their general practitioner (GP) to the CIPC counselling service for anxiety issues. Clients will be screened for GAD using the Generalised Anxiety Disorder 7-item scale (GAD-7) [33]. If a GAD diagnosis is suspected (the client’s GAD-7 score must be ≥ 11), a comprehensive assessment will follow using the Structured Clinical Interview for DSM-5, Research Version (SCID-5-RV) [34]. Clients who meet the criteria for GAD as a principal diagnosis will be assigned to one of the trial arms. To participate in the study, participants must consent to the conditions of the study (e.g., audio recording of sessions, attendance at pre- and post-therapy assessment sessions). Individuals taking psychotropic medication must be stabilised on that medication for 6 weeks prior to commencing therapy (cf. [29]). Clients receiving psychotropic medication will have to show, with their physician’s approval, a willingness to maintain this stability in medication use during the period of therapy (medication use will be monitored during the trial). Participants must also give consent for their GP to be contacted in relation to their participation in the study. Exclusion criteria are concurrent psychological treatment, suicide risk (as defined by a score other than 0 on item 16 of the Clinical Outcome in Routine Evaluation – Outcome Measure [CORE-OM] [35]: “I have made plans to end my life”), risk of harm to others (as defined by a score other than 0 on item 6 of the CORE-OM: “I have been physically violent to others”), substance abuse, psychosis, and organic brain syndrome as determined by the SCID-5-RV. For further information on intake assessment, *see below*.

### Therapists

Therapists will be trained in both EFT for GAD and CBT for GAD. All therapists will randomly deliver both conditions (*see below*), thus random allocation will be nested within therapists. Therapists will be HSE CIPC employees already working routinely within the CIPC service. Optimally all therapists will be counselling psychologists; it is planned that all therapists will have prior training in both CBT and EFT (*see below*). It is envisaged that the interventions will be delivered by at least six therapists. However, given the likelihood that therapists in CIPC may change jobs over the life of the trial (especially in the context of wider changes in recruitment practice within the HSE), we intend to train more therapists than needed to account for possible attrition.

### Interventions

#### Emotion-focused therapy for GAD

The EFT intervention follows a recently developed model [18, 19] based on a research project led by the lead author of this article, Ladislav Timulak [30]. The EFT model uses a specific case conceptualisation which postulates that rather than being avoidant of emotional experience or of emotional processing in general (as typically proposed in CBT models), clients struggle to cope with specific triggers which evoke in them specific chronic maladaptive painful feelings (e.g., sadness/loneliness, shame, and primary fear/terror). It is these triggers that the client then avoids (through emotional and behavioural avoidance, one expression of which is excessive worrying). In the context of these triggers, the client attempts to cope with his or her own painful feelings, often through negative self-treatment. It is further postulated that, contrary to mainstream CBT theories, change will be facilitated not through emotional habituation to difficult triggers, but rather through the restructuring and transformation of problematic emotion schemes through a sequence of emotional processing steps (cf. [18, 19, 36, 37]).

The model of transformation assumes that the client is first facilitated to tolerate specific painful feelings; that he or she is then facilitated to articulate the unmet needs implicit in these painful feelings; and finally that, in doing so, he or she is helped to transform these painful feelings through the generation of adaptive emotions such as compassion and healthy protective anger. The therapy focuses on (1) a firm case conceptualisation, (2) the provision of a soothing relationship, (3) the overcoming of avoidance (worry) through experiential tasks which highlight both the cost of worry and the cost of obstructing the meeting of own needs (thereby leading to a resolve to fight the worry), and (4) the transformation of chronic dreaded painful feelings through experiential tasks that generate adaptive emotions (e.g., compassion and protective anger) in response to chronic emotional pain and the unmet needs embedded in that pain.

Therapy will last 16–20 sessions. Therapists will be instructed to finish therapy at session 16, but it will be at their discretion to clinically judge whether therapy should be prolonged to a maximum of 20 sessions. This flexible ending is based on learning from the initial project [30], in which some clients needed more than the initially anticipated 16–20 sessions (in some instances up to 24 sessions). Routine practice in CIPC allows for an extension of therapy, provided there are good clinical reasons to do so. We propose that therapists continue to use the same criteria as currently used within CIPC when deciding whether to extend therapy; that is, (1) the client continues to be clinically distressed (e.g., the therapist can use a formal assessment such as the GAD-7, which is collected as part of the study), and (2) the client expresses an explicit wish to continue with therapy for up to another 4 sessions. The number of sessions will be monitored, and these data will form part of a cost-effectiveness analysis between the two active interventions.

#### Cognitive behavioural therapy for GAD

CBT models of GAD view pathological worry as central to understanding and treating GAD. These models assume that the worry process is a counterproductive avoidance mechanism through which the person tries to avoid unbearable experiences, including certain thoughts and feelings (cf. [29]). Paradoxically, worry can then directly contribute to the amount of anxiety the person actually experiences [11] and lead to unhelpful coping strategies and beliefs about the importance of controlling thoughts and worries. In Dugas and Robichaud’s CBT for GAD model [38], the main experience to be avoided is uncertainty. Worry serves as a means by which to think through possible (negative) outcomes, and possible solutions. However, given the infinite possible set of outcomes and solutions, worry does not resolve, and the increase in anxiety it produces leads instead to further thwarted attempts to problem-solve possible future scenarios.

The cognitive behavioural arm of the study will follow standard CBT procedures used in the treatment of GAD, whilst incorporating the Dugas and Robichaud model [38]. Therapy will be based on an individual case conceptualization of each participant and will involve flexibly employed standard CBT techniques such as self-monitoring, psychoeducation, cognitive restructuring, problem solving, imaginal exposure, and behavioural experiments. The therapy will aim to help clients understand the role of the mutually reinforcing relationship between intolerance of uncertainty and worry in GAD, and it will teach them to be more aware of this in their daily lives. Exposure will focus on imaginal exposure to feared outcomes and imaginal and direct exposure to uncertainty. Participants will also be encouraged to re-evaluate their beliefs around worry and to develop more active problem-solving skills. Participants will be encouraged to consider the developmental sources of GAD in order to contextualise their beliefs about worry as well as their core beliefs about themselves and the world. As with the EFT condition, therapy will last 16–20 sessions; therapists will be instructed to finish therapy at session 16 but will be allowed discretion to clinically judge whether the therapy should be prolonged to a maximum of 20 sessions.

### Therapist training and supervision

It is envisaged that all therapists will have a prior basic training in both approaches as part of their doctoral training in counselling psychology. To further boost their skills in the respective approaches, therapists will be provided with 5 days of training focused on EFT for GAD (facilitated by Ladislav Timulak, an EFT trainer accredited by the International Society for Emotion Focused Therapy) and 5 days of training in CBT for GAD (facilitated by Craig Chigwedere, course director of the master of science programme in cognitive therapy at Trinity College Dublin [TCD]). In addition, therapists will attend weekly supervision (1 week in EFT, the other in CBT) for a minimum of 8 months before being assessed as adherent to both approaches and thus released to see participants in the trial. Supervision will be provided by Ladislav Timulak for the EFT condition and by Craig Chigwedere and Charlotte Wilson (an assistant professor in clinical psychology with a special interest in CBT) for the CBT condition. Therapists must be deemed competent in the delivery of both interventions (i.e., EFT for GAD and CBT for GAD) prior to seeing participants in the trial. The decision regarding competence will be made by the supervisor(s) in the respective condition. It is intended that supervision will continue during the trial. Ten therapists have been trained, two of which have already left the study.

### Treatment fidelity assessment

In addition to on-going supervision, which will ensure therapist adherence to the respective treatment protocols, we will also conduct an independent assessment of the adherence and competence (fidelity) of the delivered treatments. All sessions will be audio-recorded. The Person-Centred and Experiential Psychotherapy Scale (EFT version) [39, 40] will be used for the EFT condition and the Cognitive Therapy Scale–Revised [41], as applied for GAD, will be used for the CBT condition. One session will be randomly selected for each case (the first two and the last two sessions from each case will not be included in the pool of potential sessions) and rated by an expert in the respective approach. Selected sessions will be rated on both scales to evaluate not just adherence but also appropriate levels of discrimination between the two approaches. To establish reliability, a portion of the sessions will be rated by at least two independent expert raters.

### Randomisation

Because the organisational structure of CIPC means that it would not always be possible to randomise between therapists (e.g., often only one therapist works in a particular location), the decision was made to randomise within therapists. David Hevey, a member of the research team with no allegiance to either intervention and no involvement in the delivery of either training or supervision, will generate the allocation sequence using an online randomiser. A random sequence (of numbers 1 and 2, corresponding to EFT or CBT) will be generated for each therapist, who will be identified by code and not by name. After SCID-5 assessment (*see below*), and after allocation to a therapist (*see* the next section on patient consent process and assessment), a member of the research team (the trial manager) will contact David Hevey and request the assignment (either EFT or CBT) for the next participant for the therapist in question.

### Patient consent process and assessment

**Fig. 1:**
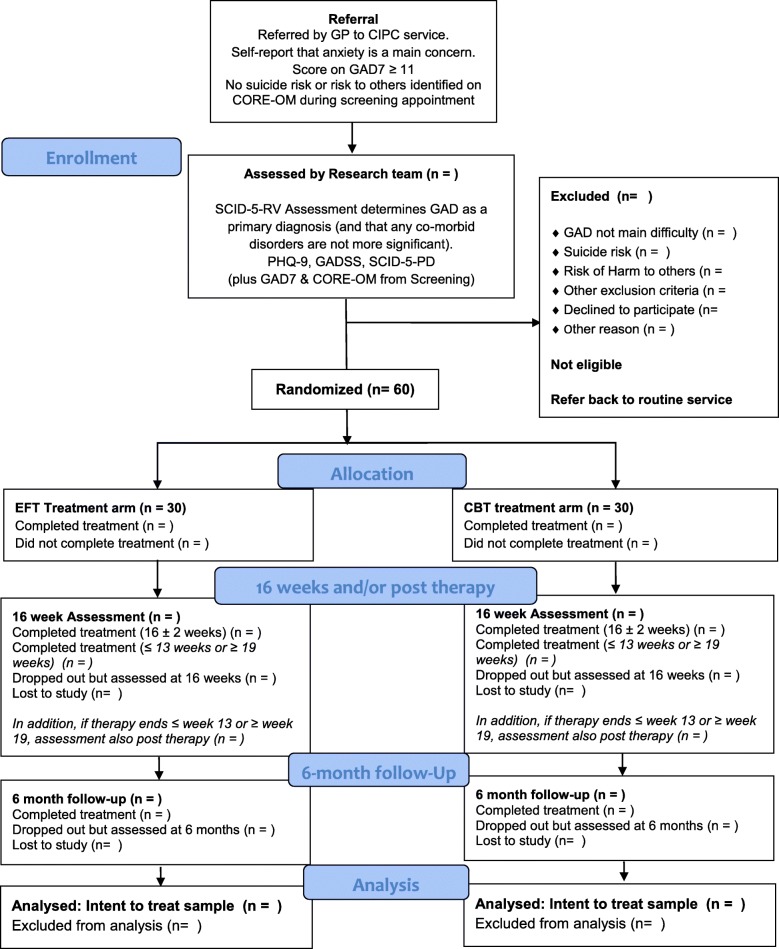
Study flow diagram of referral, screening and allocation of participants to the EFT vs CBT for GAD trial. *CBT* Cognitive behavioural therapy, *CIPC* Counselling in Primary Care, *CSS* Client Satisfaction Survey, *CORE-OM* Clinical Outcomes in Routine Evaluation – Outcome Measure, *EFT* Emotion-focused therapy, GAD Generalised anxiety disorder, *GAD-7* Generalised Anxiety Disorder-7, *GADSS* Generalised Anxiety Disorder Severity Scale, *PHQ-9* Patient Health Questionnaire-9, *SCID-5-PD* Structured Clinical Interview for DSM-5 Personality Disorders, *SCID-5-RV* Structured Clinical Interview for DSM-5 Disorders (Research Version)

Participants are recruited from among clients referred to the CIPC service. Referrals are screened centrally for suitability for the service and then allocated to CIPC therapists working in the CIPC area closest to where the client lives. The client then meets a therapist located in that area for an initial assessment. (For the flow of participants, *see* the Consolidated Standards of Reporting Trials diagram in Fig. 1). When a client presents with anxiety difficulties (as assessed by referral report and/or self-report), has a score ≥ 11 on the GAD-7, and in the assessing therapist’s clinical judgement potentially meets criteria for GAD as a principal diagnosis, the therapist will give the client information about the trial. When the client expresses an interest in participation, meets trial inclusion criteria (e.g., stabilised medication regime), and does not meet trial exclusion criteria (e.g., no current risk of suicide or risk of harm to others as measured by the CORE-OM [*see above*]), the therapist will seek client consent to refer him or her to the research team for further assessment. The therapist will provide the client with a copy of both the study information sheet and the consent form and will pass the client’s contact information to the research team. (*Note*: Administration of the CORE-OM is a routine part of the CIPC initial assessment.)

After a cooling-off period (1–2 days) the research team will contact the client to schedule an assessment appointment. At the beginning of this appointment, the research team member will discuss the study with the client, address any queries arising from their perusal of the information sheet, and seek consent to proceed with the assessment process. Assessment will involve administration of the SCID-5-RV and the Structured Clinical Interview for DSM-5 Personality Disorders (SCID-5-PD) [42]. In addition, the research team will administer the Generalised Anxiety Disorder Severity Scale (GADSS) [43] and the Patient Health Questionnaire-9 (PHQ-9) [44]. When assessment indicates GAD as a principal diagnosis, and when inclusion criteria are confirmed as being met, the client will be invited to participate in the study. Time will then be taken to address any queries the client may have, and the client will be asked to sign the study consent form.

Those individuals who proceed to become study participants will be allocated a unique trial code. They will be referred to their trial therapist and, only after allocation to a therapist, will be assigned to either the EFT or CBT condition. (Whilst typically the trial therapist will also be the therapist who initially screened the participant, in some exceptional circumstances the trial therapist may not have conducted the initial screening.)

It is anticipated that the first therapy session will typically take place 1 week after the assessment appointment with the research team. Post-treatment assessments will take place at week 16 (i.e., as close as possible to 16 calendar weeks from the date of the first session) and 6 months after 16 weeks (as close as possible to 42 calendar weeks from the date of the first session). In addition, when participants finish therapy outside the range of 16 ± 2 weeks (i.e., ≤ 13 weeks or ≥ 19 weeks), an additional assessment will be carried out post-therapy (i.e., as close as possible to the date of the last session). Sixteen-week, post-therapy and 6-month follow-up assessments will consist of administering the GAD-7, GADSS, CORE-OM and PHQ-9. In addition, the Counselling in Primary Care Client Satisfaction Survey (CIPC-CSS), which is routinely administered to all CIPC clients at the end of therapy, will be administered (*see below* for a description of measures).

If, during the course of the treatment, any client’s clinical condition suggests another course of treatment (e.g., further assessment, hospitalisation, acute risk management), this will be provided as per typical CIPC service guidelines. Fiona Ward (CIPC Director of Counselling) and the counselling co-ordinators who conduct regular clinical supervision will oversee any clinical issues arising. It has been agreed with CIPC that if, during follow-up assessment, any participant presents at risk or in significant distress, the research team will contact both CIPC and the participant’s GP to inform them of same.

### Measures

#### Screening and assessment of participants

**Fig. 2:**
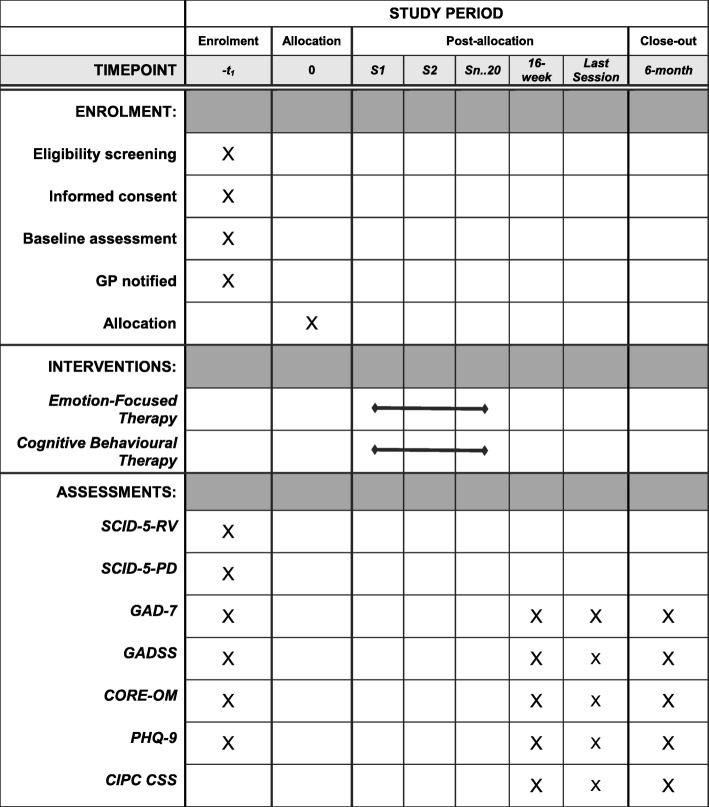
Standard Protocol Items: Recommendations for Interventional Trials (SPIRIT) diagram of enrolment, intervention and assessment. *CIPC CSS* Counselling in Primary Care Client Satisfaction Survey, *CORE-OM* Clinical Outcomes in Routine Evaluation – Outcome Measure, *GAD-7* Generalised Anxiety Disorder-7, *GADSS* Generalised Anxiety Disorder Severity Scale, *GP* General practitioner, *PHQ-9* Patient Health Questionnaire-9, *SCID-5-PD* Structured Clinical Interview for DSM-5 Personality Disorders, *SCID-5-RV* Structured Clinical Interview for DSM-5 Disorders (Research Version)

Potential participants will be screened for generalised anxiety using the GAD-7 (*see below*), with a score ≥ 11 indicating that an individual may be eligible for the study. Potential participants will then be assessed with the SCID-5-RV and the SCID-5-PD. The SCID-5-RV is a semi-structured diagnostic interview for assessing the major *Diagnostic and Statistical Manual of Mental Disorders, Fifth Edition* (DSM-5) diagnoses, while the SCID-5-PD is a semi-structured diagnostic interview for assessing the ten DSM-5 personality disorders. During assessment, demographic data (including age, gender, relationship status, living arrangements, number of dependents, level of education attained, occupation and work history, disability) as well as data related to presenting issues (including history of present and other psychological difficulties, current and past interventions, medication, substance use) will be gathered. A summary of assessments is presented in Fig. 2.

#### Primary outcome

The severity of GAD symptoms will be measured at pre-therapy, post-therapy (16 weeks; but also after the last session in cases where the last session falls outside the range of 16 ± 2 weeks) and at 6-month follow-up, using the GAD-7 as the primary outcome measure.

The GAD-7 [33] is a seven-item self-report questionnaire assessing GAD symptoms over the preceding 2 weeks. Using the threshold score of 10, the GAD-7 has been reported to have a sensitivity of 89% and a specificity of 82% for GAD; it has also been reported as having good reliability, as well as criterion, construct, factorial, and procedural validity [33].

#### Secondary outcome measures

In addition, GAD symptoms, depression symptoms and general well-being and life functioning will be measured at pre-therapy, post-therapy (16 weeks; but also after the last session in cases where the last session falls outside the range of 16 ± 2 weeks) and at 6-month follow-up using the GADSS, PHQ-9, and CORE-OM measures, respectively. In addition, the CIPC-CSS, which is routinely administered to all CIPC clients at the end of therapy, will be administered post-therapy.

CORE-OM [35] is a 34-item questionnaire designed to measure a pan-theoretical core of clients’ global distress. The CORE-OM measures psychological distress across four domains: subjective well-being, problems or symptoms, life functioning and risk. Items refer to how respondents have been feeling over the past week and are scored on a 5-point Likert scale ranging between 0 (not at all) and 4 (most or all of the time). It has been shown to have good internal and test-retest reliability, as well as good convergent validity with other measures of psychological distress [35]. The CORE-OM is routinely used in the CIPC service, from which study participants will be recruited.

The GADSS [43] is an interview rating scale designed specifically for assessing symptom severity of GAD. Respondents are asked first to identify subjects of worry (e.g., future, health, family, finances, or work). They are then asked to rate these target worries in terms of (1) frequency of worry, (2) distress due to worry, (3) frequency of associated symptoms, (4) severity and distress of associated symptom, (5) impairment in work and (6) impairment in social function. The scale has been shown to demonstrate high internal consistency (α = 0.90), good validity and sensitivity to change in a sample of adult primary care patients [43].

The PHQ-9 [44] is a nine-item self-report instrument intended to assess the existence and severity of symptoms of depression. Internal reliability of the PHQ-9 has been reported as excellent, with Cronbach’s α of 0.89 in a primary care sample, and its strong construct validity, external validity and test-retest reliability have all been satisfactory [44].

Items 10–17 of the CIPC CSS, which is routinely administered to all CIPC clients at the end of therapy, will also be analysed. Included in these items are questions asking the client how effective counselling was in helping him or her to deal with the difficulties that led him or her to seek counselling in the first place, whether he or she felt they benefited from counselling (with prompts seeking specificity regarding how it might or might not have helped), how satisfied he or she was with the counsellor (on a range of criteria, including the capacity to adopt an approach that suited the client), and how counselling came to an end.

### Assessor training

Assessments will be conducted by a doctoral-level counselling psychologist and by psychology graduates (typically at master’s level or studying at master’s level) under the supervision of the doctoral-level psychologist. SCID-5-RV, SCID-5-PD and GADSS assessments will be audio-recorded and archived for subsequent evaluation of adherence to appropriate testing conditions. Assessors conducting post-therapy assessments will be blind to the condition the client was in.

### Sample size

A sample size of 60 participants was determined (using G*Power) [45] on the basis of the comparison between the two active treatments and pre-post within-group comparison. We used a minimum meaningful comparison (moderate effect size; *f* = 0.25) that is used in assessing difference between two treatments (cf. Shapiro [46]), with statistical power of 0.80 and alpha level of 0.05.

We expect that the study is likely to detect change over time within the two active treatments (the pre-post change should be large). It is envisaged, however, that the sample size may be too small to find any difference between the active treatments (often the differences between active treatments are rather small). Because the present project is a feasibility study, exploratory use of the initial comparison data should help in planning a definite non-inferiority trial that could be developed as phase 2 of the present study, with further participants recruited to the two active interventions on the basis of an estimate of the numbers needed to determine definite evaluation.

Practically speaking, there should be a sufficient number of referrals. Annual referral numbers for CIPC Dublin North-East, where the study is hosted, is estimated at approximately 2000 clients, with at least 25% of individuals referred for anxiety problems. Because we will be recruiting over a 2-year period, we anticipate no issue with recruiting 60+ participants from a prospective pool of 1000 clients.

### Data management

All identifying paper data (i.e., signed consent forms, forms listing participant codes) will be stored in a locked file cabinet in a locked room in the School of Psychology, TCD. Each potential participant assessed by the research team will be given a unique study code which identifies the client as assessed for the study. In addition, participants who proceed from assessment to the trial will be given a unique trial code. This code will identify the client and the corresponding therapist. Paper copies of measures will be identifiable only by the study and/or trial codes. All regulations set by the ethics committees (the Research Ethics Committee at the School of Psychology, TCD, and the Research Ethics Committee, HSE, North East) as well as data protection regulations will be observed. Electronic data will be routinely cross-checked to mitigate against data entry errors, with all data double-entered into two parallel data sets, which will be routinely compared. Multiple imputation will be used for missing data. Data-monitoring oversight will be provided by David Hevey, a health psychologist and statistician with no direct allegiance to either of the two compared approaches and who will address any issues arising with the principal investigator (PI) as well as reporting directly to the trial management group (TMG) and trial steering committee (TSC) (*see below*).

### Statistical methods

The main analysis will be performed under the direction of David Hevey. The main analysis will be run as intention-to-treat analysis (within- and between-group comparisons at 16 weeks, the end of treatment, and at 6-month follow-up) as well as per-protocol analysis (which will exclude participants that had fewer than eight sessions; i.e., half of the expected length of the treatment).

Primary (GAD-7) and secondary (GADSS, PHQ-9 and CORE) outcomes will be analysed using repeated-measures analysis of variance for the two active conditions at the end of treatment, at 16 weeks, and at the 6-month follow-up. Analyses will be based on the intention-to-treat sample. Effects will be tested at the 0.05 level. The magnitude of the within-group effects of each of the interventions (Cohen’s *d*) will be calculated. The magnitude of between-group effects will be established as well. Analysis will also be conducted to determine the proportion of participants who evinced clinically significant change and reliable improvement [47] at the end of the treatment, at 16 weeks, and at 6-month follow-up for the two active treatments on the GAD-7.

### Governance and oversight of the trial

The TMG is concerned with the day-to-day operations of running the trial and will monitor all aspects of the project. The TMG will meet approximately every 2 or 3 months. Membership consists of the PI and EFT trainer and supervisor (LT); the trial manager (DK); CBT trainers and supervisors (CC and CW); CIPC Director and Director of Counselling, National Counselling Service, HSE (FW); a statistician (DH); and CIPC clinical co-ordinators (LJ and PG). The group regularly discusses issues such as training and supervision of therapists; competence and adherence to protocols; recruitment of participants; retention of participants and therapists; clinical issues that are arising; clinical governance and ethical issues; procedures for pre-therapy, post-therapy and follow up assessments; and data analysis. The TMG will also assess any adverse events or unintended effects of trial interventions or trial conduct that arise.

The TSC will meet 6-monthly over the course of the project and will have the function of independently overseeing the project. Membership will consist of an independent chairperson (with experience of conducting RCTs in a related field), the above-mentioned members of the TMG, and optimally a representative from the public. The PI will report to the committee on progress regarding the trial and seek lay/expert perspectives and queries from the group regarding any issues arising. It is intended that the group can provide an outsider perspective on issues that arise or issues that may not already have been considered by the research team. It is intended that these perspectives will be both from a scientific perspective (e.g., addressing potential research team bias) and from a lay perspective (e.g., better reflecting the experience of potential participants in the trial).

## Discussion

NICE recommends that GAD be treated with medication and/or psychological treatment, which is currently limited to various forms of CBTs [10]. Although the efficacy of CBTs is relatively well studied, questions remain about their relative efficacy and about the possibility of alternative psychological therapies [14]. Increasing treatment options appears to be desirable [16]. The present study aims to contribute to all these goals. Furthermore, there is the possibility that the study may point to the differential effects of different psychological treatments.

Specifically, the present project should further contribute to the exploration of EFT as a viable treatment for GAD. By providing first-comparison data, the present study should also contribute to the exploration of EFT as an alternative to CBT in the treatment of GAD. As a feasibility study, it should contribute to the planning of a definitive non-inferiority trial that would establish EFT’s relative efficacy in comparison to CBT as an established treatment. Given that the host of the study, the HSE’s CIPC service, is a national service employing 280 therapists and seeing several thousand clients each year, there is a clear opportunity for the present study to evolve naturally into a definitive study.

Psychotherapy research studies often show equivalence [48] with differences often in the range of 0 to 0.2 of Cohen’s *d* [49]. If findings from the present study indicate that EFT is comparable to CBT using typical psychotherapy research standards, a non-inferiority study may be a natural next step. Depending on the results of this feasibility study, we should also be able to assess the practical issues involved in running such a design with the CIPC service. We should also be able to estimate the sample size needed to run such a project.

### Trial status

Training of therapists started in January 2017, and recruitment of participants began in December 2017. Recruitment is ongoing.

## Additional file

Additional file 1: SPIRIT 2013 checklist: recommended items to address in a clinical trial protocol and related documents. (DOCX 53 kb)

## Abbreviations

CBT: Cognitive behavioural therapy
CIPC: Counselling in Primary Care
CIPC-CSS: Counselling in Primary Care – Client Satisfaction Survey
CORE-OM: Clinical Outcome in Routine Evaluation – Outcome Measure
DSM-5: *Diagnostic and Statistical Manual of Mental Disorders, Fifth Edition*
EFT: Emotion-focused therapy
GAD: Generalised anxiety disorder
GAD-7: Generalised Anxiety Disorder 7-item scale
GADSS: Generalised Anxiety Disorder Severity Scale
GP: General practitioner
HSE: Health Service Executive
NICE: National Institute for Health and Care Excellence
PHQ-9: Patient Health Questionnaire-9
PI: Principal investigator
RCT: Randomised controlled trial
SCID-5-PD: Structured Clinical Interview for DSM-5 Personality Disorders
SCID-5-RV: Structured Clinical Interview for DSM-5, Research Version
TCD: Trinity College Dublin
TMG: Trial management group
TSC: Trial steering committee
